# Alterations in Cardiac Function During Hemodynamic Fluctuations in Major Neurosurgery: An Evaluation Using Transesophageal Echocardiography

**DOI:** 10.7759/cureus.89516

**Published:** 2025-08-06

**Authors:** Nilima R Muthachen, Manikandan Sethuraman

**Affiliations:** 1 Anaesthesiology, St John's Medical College Hospital, Bangalore, IND; 2 Neuroanesthesia, Sree Chitra Tirunal Institute for Medical Sciences and Technology, Thiruvananthapuram, IND

**Keywords:** afterload, hemodynamic instability, myocardial contractility, neurosurgery, preload, transesophageal echocardiography

## Abstract

Introduction: Maintaining hemodynamic stability during the perioperative period of major neurosurgical procedures is of paramount importance. A major challenge for anesthesiologists during hemodynamic fluctuations is identifying the underlying cause to guide appropriate therapy. Limited literature is available on the utility of transesophageal echocardiography (TEE) during hemodynamic fluctuations in major neurosurgery. We prospectively investigated the role of TEE-derived measures of preload, myocardial contractility, and afterload to evaluate the causes of hemodynamic instability during neurosurgery.

Methods: Sixty-three adult patients (American Society of Anesthesiologists (ASA) risk grades 1 and 2) undergoing elective neurosurgical procedures in Sree Chitra Tirunal Institute for Medical Sciences & Technology (SCTIMST), Thiruvananthapuram, India, were included. An episode of significant hemodynamic instability was defined as a change of ±20% of either heart rate or blood pressure or both from the baseline. During each episode, TEE was used to identify changes in preload, myocardial contractility, and afterload, and these data were used to ascertain the cause of hemodynamic instability. For each of the variables, i.e., heart rate (HR), systolic blood pressure (SBP), and mean arterial pressure (MAP), a chi-square test was used to assess significant changes in the TEE variables measured between those with significant changes and those without. A p-value of <0.05 was taken as statistically significant.

Results: A total of 137 episodes of significant changes in heart rate and blood pressure were observed. Bradycardia was associated with improved preload variables. Tachycardia was associated with increased stroke volume variation (SVV), superior vena cava collapsibility index (SVC-CI), increased myocardial contractility, increased cardiac output (CO), reduced stroke volume (SV), and increased systemic vascular resistance (SVR). A decrease in SBP was associated with a decrease in preload indices. An increase in SBP showed a significant increase in SVR. A decrease in MAP was associated with preserved SVC-CI, but a significant reduction in CO. An increase in MAP was associated with a significant reduction in SV and an increase in SVR.

Conclusion: By integrating TEE measures of preload, afterload, and contractility, we gain better insight into the clinical context, which enables us to respond effectively and target therapy appropriately during the perioperative period.

## Introduction

Maintaining hemodynamic stability is one of the key goals in major neurosurgical procedures. However, severe hemodynamic fluctuations are often seen during the intraoperative period, which can impact both neurological and systemic outcomes [1,2,3,4]. Intraoperative management strategies to maintain stable hemodynamics significantly impact long-term outcomes, influencing postoperative metabolic status and renal function [5]. Management of hemodynamic fluctuations is a major challenge for anesthesiologists, who rely on the hemodynamic variables from intraoperative monitors to identify the causes and target therapy [6,7]. Currently, the management of intraoperative hemodynamic instability often starts with volume loading for hypotension, with vasopressors added if necessary. For hypertension, treatment typically involves opioid analgesics and increasing the depth of anesthesia. Vasodilators are generally avoided due to their potential to raise intracranial pressure (ICP). This empirical approach can lead to treatment delays and failures.

Goal-directed fluid therapy (GDT) is another useful technique for managing hemodynamic fluctuations in neurosurgery [8]. However, a recent meta-analysis found no difference in outcomes, including incidence of hypotension, in GDT versus conventional therapy [9]. The utility of intraoperative transesophageal echocardiography (TEE) has been well established in major vascular and cardiac procedures [10,11]. However, limited literature exists regarding the use of TEE-based hemodynamic management in non-cardiac surgeries like neurosurgery. TEE provides more comprehensive information, particularly regarding myocardial performance, compared to conventional monitoring and non-invasive advanced cardiac monitors in the context of hemodynamic disturbances [12,13].

The use of echocardiography is generally recommended in patients with hemodynamic instability who fail to respond to conventional management. We hypothesized that hemodynamic instability occurring during the intraoperative period of neurosurgery is associated with changes in more than one factor, compared to traditional teaching and management using fluids. The use of TEE intraoperatively would aid in the estimation of severity and the etiology of the hemodynamic instability more accurately than conventional monitoring methods.

The primary aim of this study is to assess the effects of intraoperative hemodynamic instability during neurosurgical procedures on TEE-derived measures of preload, myocardial contractility, and afterload. The secondary aim is to identify the TEE variables that may provide more reliable indicators than conventional heart rate and continuous arterial blood pressure for managing hemodynamic instability during neurosurgery.

This article was previously posted to the SCTIMST DSpace (digital repository of Sree Chitra Tirunal Institute for Medical Sciences and Technology (SCTIMST)) on January 23, 2017.

## Materials and methods

The study was planned as a prospective pilot observational study. The study was approved by the Institutional Ethics Committee of SCTIMST, Thiruvananthapuram, India (IEC Regn. No. ECR/189/Inst/KL/2013). Patients undergoing elective major neurosurgery for supratentorial pathology at SCTIMST were enrolled between June 2015 and May 2016, after obtaining informed written consent. Adult patients aged >18 years, American Society of Anesthesiologists (ASA) risk grade of 1 or 2, Glasgow coma scale score of 14 or 15, undergoing elective major neurosurgical procedures (duration >6 hours) with anticipated hemodynamic disturbances, significant blood loss (>10%), large tumors (size >6 cm) on preoperative radiological investigations or an increased risk for intraoperative venous air embolism for whom the use of TEE was considered beneficial, were included in the study. We excluded patients with preoperative uncontrolled hypertension, cardiac dysfunction, poor nutritional status, electrolyte and endocrine disturbances, severely raised intracranial pressure, low GCS (<13), ASA Grade 3 and 4, patient refusal, and any absolute and relative contraindication to placing the TEE probe. Preoperative fasting guidelines as per institutional protocol were followed. In the operating room, baseline hemodynamic variables, including heart rate (HR), systolic blood pressure (SBP), mean arterial pressure (MAP), and oxygen saturation, were recorded. A radial arterial line was inserted under local anesthesia prior to the induction of general anesthesia. Baseline systolic blood pressure was recorded from the arterial line before the administration of anesthetic agents and the initiation of positive pressure ventilation. General anesthesia was induced with intravenous propofol (2mg/kg) and fentanyl (2 mcg/kg), and muscle relaxation was achieved with intravenous vecuronium (0.2 mg/kg). All patients were mechanically ventilated in volume-controlled mode with a tidal volume of 8 ml/kg and a respiratory rate of 10-12 per minute, targeting an end-tidal carbon dioxide of 32-35 mm Hg and a PEEP of 5 cm of H_2_O. After endotracheal intubation, an adult TEE probe (GE Vivid 7 dimension, USA) was inserted.

Baseline recordings of the following TEE-derived variables were made: right ventricular preload, including the superior vena cava collapsibility index (SVC-CI) and inferior vena cava collapsibility index (IVC-CI); left ventricular preload, including left ventricular end-diastolic volume variation (LVEDV), stroke volume variation (SVV), and diastolic E/E' ratio; right ventricular contractility as measured by tricuspid annular plane systolic excursion (TAPSE); left ventricular contractility, including ejection fraction (EF), cardiac output (CO), stroke volume (SV), and regional wall motion abnormalities (RWMA); and left ventricular afterload, assessed by systemic vascular resistance (SVR), derived from cardiac output and mean arterial pressure. Changes in TEE parameters were classified as "No change" if they remained within ±10% of the baseline value, as "Decrease" if they declined by more than 10% below baseline, and as "Increase" if they exceeded 10% above baseline.

An episode of significant hemodynamic instability was defined as a change of ±20% in either heart rate, arterial blood pressure, or both from the baseline. During each episode of hemodynamic instability, TEE was immediately used to identify the changes and ascertain the cause of hemodynamic instability. The surgical steps (pinning, positioning, skin- to -dura, dissection of tumor, or closure). and anesthetic phases (induction, maintenance, and emergence) at the time of each reading were recorded. Any untoward event, such as venous air embolism or paradoxical air embolism, was also recorded. During the surgical procedure, TEE-derived values were recorded randomly in patients without significant hemodynamic instability. For comparison of the TEE data during hemodynamic changes, analysis was done using TEE-derived variables of preload, myocardial contractility, and afterload during episodes of hemodynamic instability with baseline as compared to those who did not have any instability. Sample size could not be calculated because the literature search did not reveal similar studies, as well as the true incidence of significant hemodynamic changes occurring intraoperatively in elective supratentorial surgeries

Statistical analysis

Statistical analysis was done using IBM SPSS Statistics for Windows, Version 21.0 (released 2012, IBM Corp., Armonk, NY). The demographics and hemodynamic variables were expressed as mean ± SD. Comparison was made between episodes with hemodynamic instability and without for each variable of preload, afterload, and myocardial contractility. For each variable (HR, SBP, and MAP), the chi-square test was used to assess significant changes in the TEE variables measured between those with significant change and those without. Chi-square test or Fisher's exact test was used to test the association between TEE-derived variables and hemodynamic changes. A p-value of <0.05 was taken as statistically significant.

## Results

We recruited 63 patients (34 males and 29 females) during the study period. The mean age was 42 ± 11 years, the mean weight was 61 ± 8 kg, and the mean height was 161 ± 7 cm (Table 1). The etiology of the neurosurgical cases (Table 2) and baseline hemodynamic measurements (Table 3) are provided.

**Table 1:** Demographic characteristics of the study participants kg: kilogram, cm: centimeters, m: meter, BSA: body surface area, SD: standard deviation

Demographics	Mean ± SD
Age (years)	42 ± 11
Weight (kg)	61 ± 8
Height (cm)	161 ± 7
BSA (m^2^)	1.64 ± 0.14
Male:female	34:29

**Table 2:** Distribution details of the various surgical procedures performed in the included study participants

Surgical procedure etiology	Number (total = 63)	Percentage
Brain tumour decompression	43	68.3
Epilepsy surgery	8	12.7
Aneurysm clipping	8	12.7
Excision of arteriovenous malformation (AVM)	3	4.7
Decompression for abscess	1	1.6

**Table 3:** Baseline heart rate, systolic blood pressure, and mean arterial pressure of the study participants HR: heart rate, SBP: systolic blood pressure, MAP: mean arterial pressure, SD: standard deviation, min: minute,mmHg: millimeters of Mercury

Parameter	Mean ± SD
Mean HR (beats/min)	69.71 ± 11.19
Mean SBP (mmHg)	121.16 ± 10.24
Mean MAP (mmHg)	86.4 ± 9.76

No complications related to the use of the TEE probe were observed in our study. Significant hemodynamic changes occurred in 36 (57%) out of 63 patients. Twenty-nine patients had more than two episodes of hemodynamic fluctuation. In a total of 137 episodes of hemodynamic instability, the significant events included tachycardia (27 episodes, 20.5%), bradycardia (five episodes, 4.5%), an increase in SBP (10 episodes, 8.5%), a decrease in SBP (20 episodes, 15.7%), an increase in MAP (19 episodes, 16.4%), and a decrease in MAP (21 episodes, 17.8%) (Table 4). None of the patients in the study exhibited abnormal tricuspid annular plane systolic excursion (TAPSE) values or regional wall motion abnormalities (RWMA) on TEE.

**Table 4:** Number of episodes of significant hemodynamic changes in study participants intraoperatively: distribution of variations in heart rate (HR), systolic blood pressure (SBP), and mean arterial pressure (MAP)

Hemodynamic variable	+20% increase	-20% decrease	No change
Heart rate changes	27	5	105
Systolic blood pressure changes	10	20	107
Mean arterial pressure changes	19	21	97

In our study, the incidence of significant bradycardia (4.5%) was low, and none of the TEE variables related to preload, contractility, or afterload were significantly different compared to those without bradycardia (Tables 5, 6).

**Table 5:** Bradycardia with preload parameters TEE: transesophageal echocardiography, SVC-CI: superior vena cava collapsibility index, IVC-CI: inferior vena cava collapsibility index, EDV: left ventricular end diastolic volume variation, E/E’: diastolic E/E’ ratio, SVV: stroke volume variation (Fisher's exact test (p < 0.05 significant)) Percentages in brackets

TEE-derived variables	No change in heart rate (N = 105)	Bradycardia (N = 5)	p
SVC-CI increase	42 (40)	1 (20)	0.298
SVC-CI decrease	47 (45)	2 (40)	
SVC -CI no change	15 (14)	2 (40)	
IVC-CI increase	34 (32.4)	0	0.108
IVC- CI decrease	48 (46)	5 (100)	
IVC -CI no change	17 (16.2)	0	
EDV increase	27 (25.7)	1 (20)	0.710
EDV decrease	51 (48.6)	2 (40)	
EDV no change	23 (22)	2 (40)	
E/E’ increase	41 (39)	2 (40)	1.000
E/E’ decrease	35 (33)	2 (40)	
E/E’ no change	25 (24)	1 (20)	
SVV increase	26 (25)	1 (20)	1.000
SVV decrease	44 (42)	3 (60)	
SVV no change	29 (26.7)	1 (20)	

**Table 6:** Bradycardia with contractility and afterload parameters TEE: transesophageal echocardiography, CO: cardiac output, SV: stroke volume, EF: ejection fraction, SVR: systemic vascular resistance (Fisher's exact test (p < 0.05 significant)) Percentages in brackets

TEE-derived variables	No change in HR (N = 105)	Bradycardia (N = 5)	p
CO increase	39 (37)	2 (40)	1.000
CO decrease	39 (37)	2 (40)	
CO no change	25 (24)	1 (20)	
SV increase	34 (32.4)	1 (20)	0.617
SV decrease	35 (33.3)	3 (60)	
SV no change	34 (32.4)	1 (20)	
EF increase	22 (21)	1 (20)	1.000
EF decrease	41 (39)	2 (40)	
EF No change	40 (38)	2 (40)	
SVR increase	34 (32)	1 (20)	0.849
SVR decrease	46 (44)	3 (60)	
SVR-No change	22 (21)	1 (20)	

During significant tachycardia episodes, it was noted that both right (increased SVC-CI) and left-sided (low EDV, decreased E/E’, increased SVV) preload were low, myocardial contractility was increased (increased EF), and there was a higher percentage of afterload changes compared to those without changes (Tables 7, 8).

**Table 7:** Tachycardia with preload parameters TEE: transesophageal echocardiography, SVC-CI: superior vena cava collapsibility index, IVC-CI: inferior vena cava collapsibility index, EDV: left ventricular end diastolic volume variation, E/E’: diastolic E/E’ ratio, SVV: stroke volume variation (Fisher's exact test (p < 0.05 significant)) Percentage in brackets

TEE-derived variables	No change in heart rate (N = 105)	Tachycardia (N = 27)	p
SVC-CI increase	42 (40)	15 (55.6)	0.358
SVC-CI decrease	47 (45)	10 (37)	
SVC -CI no change	15 (14)	2 (7.4)	
IVC-CI increase	34 (32)	9 (33.3)	0.737
IVC-CI decrease	48 (45.7)	11 (40)	
IVC -CI no change	17 (16.2)	6 (22.2)	
EDV increase	27 (26)	7 (26)	0.654
EDV decrease	51 (48.6)	16 (59)	
EDV no change	23 (22)	4 (15)	
E/E’ increase	41 (39)	9 (33)	0.141
E/E’ decrease	35 (33.3)	15 (56)	
E/E’ no change	25 (24)	3 (11)	
SVV increase	26 (25)	10 (38.5)	0.033
SVV decrease	44 (42)	4 (15)	
SVV no change	29 (26.7)	10 (38.5)	

**Table 8:** Tachycardia with contractility and afterload parameters TEE: transesophageal echocardiography, CO: cardiac output, SV: stroke volume, EF: ejection fraction, SVR: systemic vascular resistance (Fisher's exact test (p < 0.05 significant) used for all values except CO, for which Pearson's chi-square test (p < 0.05 significant, test statistic 0.6611) was used) Percentages in brackets

TEE-derived variables	No change in HR (N = 105)	Tachycardia (N = 27)	p
CO increase	39 (37)	12 (45)	0.719
CO decrease	39 (37)	8 (30)	
CO no change	25 (24)	7 (26)	
SV increase	34 (32.4)	4 (15)	0.152
SV decrease	35 (33.3)	13 (48)	
SV no change	34 (32.4)	10 (37)	
EF increase	22 (21)	11 (40)	0.061
EF decrease	41 (39)	11 (40)	
EF No change	40 (38)	5 (18.5)	
SVR increase	34 (32)	11 (40.7)	0.816
SVR decrease	46 (44)	11 (40.7)	
SVR-No change	22 (21)	5 (18.5)	

A decrease in systolic blood pressure (SBP) was predominantly associated with effects on the left-sided heart, including a decrease in left-sided preload indices (reduced LVEDV, E/E’ ratio, increased SVV), myocardial contractility (lower ejection fraction), and lower afterload (SVR). By contrast, right-sided indices were not significantly affected compared to those with lower SBP (Tables 9, 10).

**Table 9:** Decrease in systolic blood pressure with preload parameters TEE: transesophageal echocardiography, SBP: systolic blood pressure, SVC-CI: superior vena cava collapsibility index, IVC-CI: inferior vena cava collapsibility index, EDV: left ventricular end diastolic volume variation, E/E’: diastolic E/E’ ratio, SVV: stroke volume variation (Fisher's exact test (p < 0.05 significant)) Percentage in brackets

TEE-derived variables	No change in SBP (N = 107)	Decrease in SBP (N = 20)	p
SVC-CI increase	47 (44)	4 (20)	0.062
SVC-CI decrease	47 (44)	11 (55)	
SVC -CI no change	12 (11)	5 (25)	
IVC-CI increase	33 (31)	5 (25)	0.127
IVC-CI decrease	47 (44)	14 (70)	
IVC -CI no change	20 (18.5)	1 (5)	
EDV increase	26 (24.3)	5 (25)	0.321
EDV decrease	51 (47.7)	13 (65)	
EDV no change	26 (24.3)	2 (10)	
E/E’ increase	46 (43)	5 (25)	0.235
E/E’ decrease	37 (35)	9 (45)	
E/E’ no change	20 (18)	6 (30)	
SVV increase	26 (24.3)	6 (30)	0.242
SVV decrease	44 (41)	5 (25)	
SVV no change	29 (27)	9 (45)	

**Table 10:** Decrease in systolic blood pressure with contractility and afterload parameters TEE: transesophageal echocardiography, SBP: systolic blood pressure, CO: cardiac output, SV: stroke volume, EF: ejection fraction, SVR: systemic vascular resistance (Fisher's exact test (p < 0.05 significant)) Percentages in brackets

TEE-derived variables	No change in SBP (N = 107)	Decrease in SBP (N = 20)	p
CO increase	38 (35.5)	11 (55)	0.162
CO decrease	42 (39)	4 (20)	
CO no change	25 (23.4)	5 (25)	
SV increase	27 (25)	8 (40)	0.240
SV decrease	38 (35.5)	8 (40)	
SV no change	40 (37.4)	4 (20)	
EF increase	29 (27)	2 (10)	0.053
EF decrease	38 (35.5)	13 (65)	
EF No change	38 (35.5)	5 (25)	
SVR increase	37 (34.6)	0	<0.001
SVR decrease	39 (36.4)	20 (100)	
SVR-No change	28 (26)	0	

An increase in SBP was not associated with a significant change in SVC-CI or IVC-CI in preload and myocardial contractility. The only significant change was an increase in SVR (Tables 11, 12). 

**Table 11:** Increase in systolic blood pressure with preload parameters TEE: transesophageal echocardiography, SBP: systolic blood pressure, SVC-CI: superior vena cava collapsibility index, IVC-CI: inferior vena cava collapsibility index, EDV: left ventricular end diastolic volume variation, E/E’: diastolic E/E’ ratio, SVV: stroke volume variation (Fisher's exact test (p < 0.05 significant)) Percentages in brackets

TEE-derived variables	No change in SBP (N = 107)	Increase in SBP (N = 10)	P
SVC-CI increase	47 (43.9)	7 (70)	0.122
SVC-CI decrease	47 (43.9)	1 (10)	
SVC -CI no change	12 (11)	1 (10)	
IVC-CI increase	33 (31)	5 (50)	0.470
IVC-CI decrease	47 (44)	3 (30)	
IVC -CI no change	20 (18.7)	2 (20)	
EDV increase	26 (24.3)	4 (40)	0.524
EDV decrease	51 (47.7)	5 (50)	
EDV no change	26 (24.3)	1 (10)	
E/E’ increase	46 (43)	1 (10)	0.073
E/E’ decrease	37 (35)	6 (60)	
E/E’ no change	20 (18.7)	3 (30)	
SVV increase	26 (24.3)	2 (20)	0.908
SVV decrease	44 (41)	5 (50)	
SVV no change	29 (27)	2 (20)	

**Table 12:** Increase in systolic blood pressure with contractility and afterload parameters TEE: transesophageal echocardiography, SBP: systolic blood pressure, CO: cardiac output, SV: stroke volume, EF: ejection fraction, SVR: systemic vascular resistance (Fisher's exact test (p < 0.05 significant)) Percentage in brackets

TEE-derived variables	No change in SBP (N = 107)	Increase in SBP (N = 10)	p
CO increase	38 (35.5)	4 (40)	0.769
CO decrease	42 (39)	3 (30)	
CO no change	25 (23.4)	3 (30)	
SV increase	27 (25)	4 (40)	0.190
SV decrease	38 (35.5)	5 (50)	
SV no change	40 (37.4)	1 (10)	
EF increase	29 (27)	3 (30)	0.190
EF decrease	38 (35.5)	3 (30)	
EF No change	38 (35.5)	4 (40)	
SVR increase	37 (34.6)	9 (90)	0.004
SVR decrease	39 (36.4)	1 (10)	
SVR-No change	28 (26)	0	

A decrease in mean arterial pressure (MAP) was associated with non-significant changes in preload indices, but a significant reduction in cardiac output, low LVEDV, and low SVR (Tables 13, 14).

**Table 13:** Decrease in mean arterial pressure with preload parameters TEE: transesophageal echocardiography, MAP: mean arterial pressure, SVC-CI: superior vena cava collapsibility index, IVC-CI: inferior vena cava collapsibility index, EDV: left ventricular end diastolic volume variation, E/E’: diastolic E/E’ ratio, SVV: stroke volume variation (Fisher's exact test (p <0.05 significant)) Percentage in brackets

TEE-derived variables	No change in MAP (N = 97)	Decrease in MAP (N = 21)	p
SVC-CI increase	38 (39)	7 (33)	0.023
SVC-CI decrease	49 (50)	7 (33)	
SVC -CI no change	9 (9.3)	7 (33)	
IVC-CI increase	30 (31)	5 (24)	0.021
IVC-CI decrease	43 (44.3)	16 (76)	
IVC -CI no change	17 (17.5)	0	
EDV increase	24 (24.7)	5 (24)	0.949
EDV decrease	47 (48.5)	12 (57)	
EDV no change	22 (22.7)	4 (19)	
E/E’ increase	45 (46.4)	4 (19)	0.009
E/E’ decrease	34 (35)	8 (38)	
E/E’ no change	14 (14.4)	9 (43)	
SVV increase	24 (24)	6 (28.6)	0.54
SVV decrease	40 (40)	6 (28.6)	
SVV no change	28 (28)	8 (38)	

**Table 14:** Decrease in mean arterial pressure with contractility and afterload parameters TEE: transesophageal echocardiography, MAP: mean arterial pressure, CO: cardiac output, SV: stroke volume, EF: ejection fraction, SVR: systemic vascular resistance (Fisher's exact test (p < 0.05 significant)) Percentages in brackets

TEE-derived variables	No change in MAP (N = 97)	MAP decrease (N = 21)	p
CO increase	35 (36)	8 (38)	0.787
CO decrease	35 (36)	9 (43)	
CO no change	25 (26)	4 (19)	
SV increase	24 (24.7)	8 (38)	0.176
SV decrease	34 (35)	9 (43)	
SV no change	37 (38)	4 (19)	
EF increase	27 (27.8)	2 (9.5)	0.068
EF decrease	34 (35)	13 (62)	
EF No change	34 (35)	6(28)	
SVR increase	32 (33)	2(9.5)	0.007
SVR decrease	39 (40.2)	17(81)	
SVR-No change	23 (23.7)	2(9.5)	

An increase in MAP was associated with an increase in SVC and IVC-CI, reduced LVEDV, and a reduction in E/E’; however, there was a significant reduction in SV and an increase in SVR (Tables 15, 16).

**Table 15:** Increase in mean arterial pressure with preload parameters TEE: transesophageal echocardiography, MAP: mean arterial pressure, SVC-CI: superior vena cava collapsibility index, IVC-CI: inferior vena cava collapsibility index, EDV: left ventricular end diastolic volume variation, E/E’: diastolic E/E’ ratio, SVV: stroke volume variation (Fisher's exact test (p < 0.05 significant)) Percentage in brackets

TEE-derived variables	No change in MAP (N = 97)	Increase in MAP (N = 19)	p
SVC-CI increase	38 (39)	13 (69)	0.012
SVC-CI decrease	49 (50.5)	3 (16)	
SVC -CI no change	9 (9.3)	3 (16)	
IVC-CI increase	30 (31)	8 (42)	0.190
IVC-CI decrease	43 (44.3)	5 (26)	
IVC -CI no change	17 (17.5)	6 (31)	
EDV increase	24 (24.7)	6 (31.6)	0.714
EDV decrease	47 (48.5)	10 (53)	
EDV no change	22 (22.7)	3 (16)	
E/E’ increase	45 (46.4)	3 (16)	0.017
E/E’ decrease	34 (35)	10 (53)	
E/E’ no change	14 (14.4)	6 (31.6)	
SVV increase	24 (24)	8 (42)	0.294
SVV decrease	40 (40)	5 (26)	
SVV no change	28 (28)	4 (21)	

**Table 16:** Increase in mean arterial pressure with contractility and afterload parameters TEE: transesophageal echocardiography, MAP: mean arterial pressure, CO: cardiac output, SV: stroke volume, EF: ejection fraction, SVR: systemic vascular resistance (Fisher's exact test (p < 0.05 significant)) Percentage in brackets

TEE-derived variables	No change (N = 97)	Increase in MAP (N = 19)	p
CO increase	35 (36)	10 (52)	0.507
CO decrease	35 (36)	5 (26)	
CO no change	25 (26)	4 (21)	
SV increase	24 (24.7)	7 (37)	0.336
SV decrease	34 (35)	8 (42)	
SV no change	37 (38)	4 (21)	
EF increase	27 (27.8)	5 (26)	1.000
EF decrease	34 (35)	7 (37)	
EF no change	34 (35)	7 (37)	
SVR increase	32 (33)	12 (63)	0.155
SVR decrease	39 (40.2)	4 (21)	
SVR-no change	23 (23.7)	3 (16)	

## Discussion

In the present study, our aim was to assess changes in echocardiographic indices during significant hemodynamic disturbances that can occur in non-cardiac surgeries, such as neurosurgery, using TEE. We noted that significant hemodynamic episodes occurred in 57% of cases, with tachycardia being more common. Another significant finding in our study is that multiple indices were altered in patients with hemodynamic perturbations compared to those without such disturbances. There is a wide variation in the intraoperative pattern of TEE indices between hemodynamically stable and altered patients. The degree of severity and the combination of indices of preload, left versus right side filling, myocardial contractility, and afterload are likely relevant to the clinical manifestations of hemodynamic changes.

Current practices in the perioperative management of patients, with the goal of maintaining stable hemodynamics, rely on the assessment of both static and dynamic indices. Based on these changes, goal-directed fluid therapy is administered under the assumption that alterations in preload are the primary cause [14]. However, in patients with low SBP, we observed that a greater number of patients exhibited low left-sided preload, contractility, and afterload, while changes in right-sided preload were not as prominent. Hence, for appropriate management, all the factors need to be considered.

There were no statistically significant changes observed in the TEE-derived variables in relation to variations in heart rate, including both bradycardia and tachycardia. In our study, patients had more tachycardia (20.5%) than bradycardia (4.5%). The commonly recognized causes of tachycardia under anesthesia are hypovolemia, blood loss, a lighter plane of anesthesia or pain, and increased sympathetic stimulation, among others. The expected TEE changes in hypovolemia include a reduction in preload, decreased ventricular filling, low SV, decreased CO, and a compensatory increase in SVR. By contrast, increased sympathetic discharge can lead to an increase in CO and SVR. This may be a compensatory response to hypovolemia or lighter plane, or inadequate anesthesia. Without recognizing the cause of tachycardia, increasing the depth of anesthesia or administering analgesics in tachycardia can lead to worsening in a hypovolemic patient. On analysis, we found that in tachycardia, there were more patients with increased SVC-CI, increased SVV, low LVEDV, low E/E’, preserved SVR, and decreased SV compared to patients with no change in heart rate. The preserved SVR and CO could be due to increased sympathetic activity. Hence, our study showed that patients with tachycardia exhibited TEE features of reduced right ventricular and left ventricular preload with compensation in CO and SVR. Myocardial contractility was well preserved. In such patients, administering rate-controlling drugs, increasing anesthesia depth, or using opioids may be harmful if the underlying hypovolemia is not corrected. However, excessive fluid administration may also be detrimental. TEE parameters can aid in assessing the amount of fluid required.

Patients with bradycardia showed no significant changes in TEE-derived variables compared to those without heart rate changes, likely due to the transient nature of bradycardia and the small sample size, which limited the ability to identify trends.

In patients with low SBP, there were no significant differences in preload indices like SVC and IVC collapsibility, SVV, and E/E’, along with reduced myocardial contractility (decreased EF) and a significant reduction in SVR. This indicates that these patients had a significantly low afterload and reduced myocardial contractility with a well-preserved preload as a cause of low SBP. In patients with low intraoperative MAP, we found a statistically significant decrease in SVR with preserved SVC collapsibility, SVV, and E/E’ compared to those without a decrease in MAP. This suggests that low MAP was due to significantly reduced afterload while preload remained well preserved. This could be attributed to the effects of a deeper plane of anesthesia or peripheral vasodilation caused by various drugs. Administration of vasopressors or inotropes would be a more appropriate management strategy than volume loading, which may be detrimental in patients with reduced cardiac function.

In patients with increased MAP, we observed features of low preload (increased SVC-CI and IVC-CI), increased contractility (elevated CO), and a statistically significant increase in SVR. Administration of intravenous fluids would be more beneficial in this situation than increasing the depth of anesthesia, as vasodilators are harmful in patients with raised intracranial pressures. Patients with increased SBP also exhibited increased SVR (p = 0.008), along with features of low preload (SVC-CI, IVC-CI) and increased contractility parameters, compared to patients with no change.

SVV was identified as a good predictor of preload. Berkenstadt et al. studied 15 neurosurgical patients and found that responders and nonresponders had similar pre-volume loading HR and CVP values, but differed in SVV with a specificity of 93% [15]. Cannesson et al. studied the SVV in 25 patients undergoing coronary artery bypass grafting (CABG), finding that SVV was significantly higher in responders compared to non-responders (15 ± 5% vs. 7 ± 4%, respectively; P < 0.01) [16]. In the study by Khwannimit et al., the SVV was found to predict fluid responsiveness in 42 passively ventilated patients with septic shock [17]. In our study, increased heart rate episodes were linked to decreased preload indicators, such as elevated SVV and SVC-CI, although these findings were not statistically significant.

Feissel et al. studied the response of CO and IVC-CI to a standardized volume load in 39 critically ill, mechanically ventilated patients. Those who showed an increase in CO had a higher baseline IVC-CI compared to non-responders [18]. Sefidbakht et al. found a higher vena cava collapsibility index in shock patients. In a study of 88 trauma patients, the shock group had a significantly higher mean collapsibility index than the control group (27% vs. 20%, p < 0.001) [19]. These studies highlight how the vena cava collapsibility index can be used to detect volume-responsive patients. In our study, we found that in episodes with tachycardia, there was a higher number of episodes with increased SVC-CI (55.6% of episodes with increased SVC-CI vs. 37% with decreased SVC-CI).

Global end-diastolic volume is a reliable indicator of cardiac preload [20], as demonstrated by Renner et al. [21] and Michard et al. [22]. In our study, 59% of tachycardia episodes were associated with decreased end-diastolic volume, suggesting that tachycardia may have been caused by hypovolemia. However, fluid responsiveness was not assessed, as it fell outside the scope of our study.

We observed a statistically significant correlation between blood pressure and SVR. A decrease in SBP was significantly associated with reductions in SVR (100% of episodes, p = 0). Notably, cardiac output increased despite the decrease in SBP, possibly due to the associated drop in SVR. An increase in SBP was significantly correlated with an increase in SVR (90% of episodes, p = 0.008). In addition, decreases in MAP were significantly linked to reductions in SVR (81% of episodes, p = 0.009). Although episodes of increased MAP were associated with an increase in SVR (63% of episodes), this finding was not statistically significant.

Intraoperative drops in blood pressure are commonly attributed to hypovolemia caused by dehydration or blood loss. However, our study found that a decrease in SVR was more frequently associated with the observed reductions in blood pressure. Blood pressure management should be guided by the underlying cause, as factors such as anesthetic agents, hypovolemia, surgical position, mechanical ventilation, and cardiac conditions can all contribute to similar changes [23]. A decrease in pressure due to reduced volume status warrants fluid administration, while a decline in SVR requires the use of vasopressors.

Walsh et al. analyzed data from 33,330 noncardiac surgeries and found that even brief episodes of intraoperative hypotension (MAP < 55 mmHg) were linked to postoperative acute kidney injury and myocardial ischemia [24]. Kheterpal et al. analyzed hemodynamic data from 7,740 patients undergoing non-cardiac surgery to identify risk factors for cardiac adverse events (CAE). They found that high-risk patients with CAEs were more likely to experience hypotension (MAP < 50 mmHg or a 40% decrease) or tachycardia (heart rate > 100 beats per minute) [25]. This emphasizes the value of using TEE to accurately identify the causes of hypotension and guide tailored treatment, rather than relying on empirical methods.

TEE is increasingly utilized in noncardiac surgery to identify regional wall motion abnormalities indicative of myocardial ischemia [26,27]. However, in our study, we did not observe any instances of RWMA in our patients.

Our study has a few limitations. As a first-of-its-kind pilot study, the sample size may be small. Since this was an observational study, we did not directly assess the impact of therapeutic interventions based on TEE readings. Despite the maintenance of hemodynamics, many patients showed alterations in TEE indices from baseline. Hence, reliable indices for predicting and preventing hemodynamic changes may be difficult. Our focus was restricted to elective neurosurgical procedures; however, future research could broaden the application of TEE to include emergency surgeries, trauma cases, and the evaluation of patients in critical care settings. In addition, we did not examine a single variable that could predict changes in hemodynamics, as multiple factors were found to influence these changes.

## Conclusions

Our study provides insights into the hemodynamic challenges commonly encountered by anesthesiologists in neurosurgery and evaluates the role of the TEE-guided approach in identifying variables that aid in managing hemodynamic instability. No single variable can fully quantify preload, contractility, or afterload; rather, it is the combination of measured parameters and their interactions that provides clarity in clinical decision-making. Conventional clinical indicators, such as heart rate and blood pressure, can be misleading; for instance, tachycardia may result from pain, anxiety, fever, or reduced volume status. Integrating TEE measurements of preload, afterload, and contractility offers a more comprehensive understanding of the clinical context, facilitating more effective interventions and targeted therapy during the perioperative period.
